# PARTICIPANT ACCEPTABILITY OF THE DEPRESSION AND PAIN PERSEVERANCE THROUGH EMPOWERED RECOVERY PROGRAM

**DOI:** 10.1093/geroni/igad104.0685

**Published:** 2023-12-21

**Authors:** Catherine Clair, Janiece Taylor, Rebekah Bhansali, Tracie Harrison, Anna Beeber, Sarah L Szanton

**Affiliations:** Johns Hopkins Bloomberg School of Public Health, Baltimore, Maryland, United States; Johns Hopkins University, Baltimore, Maryland, United States; Johns Hopkins School of Nursing, Baltimore, Maryland, United States; The University of Arkansas for Medical Sciences, Little Rock, Arkansas, United States; Johns Hopkins University, Baltimore, Maryland, United States; Johns Hopkins University, Baltimore, Maryland, United States

## Abstract

Between 2019-2023, the Depression and Pain Perseverance through Empowered Recovery (DAPPER) program was implemented with older African American women living with pain and low mood (N=34) in Baltimore, MD. Of the 21 who completed the intervention, we interviewed 9 women to understand their experiences in the program, their successes, challenges, and areas to improve the program. The average age of the participants was 64.8 (SD: 10.5). Using a qualitative descriptive design, we analyzed the interviews using content analysis. Overall, the main themes related to 1) Changes in Pain Experiences; 2) Influence of Research Study Team; and 3) Value in Resources and Being Listened To. The women described their experience in the DAPPER program positively and the impact that it had on their pain experience. Additionally, participants mentioned how the qualities, knowledge, and perspectives of the study team impacted them: “Respectful,” “A good listener,” and “Caring.” Finally, several of the women mentioned the value of strategies learned, resources provided, and the impact that communicating with the nurses had on them. These findings demonstrate the acceptability of the DAPPER program and highlight the importance of innovative components of intervention and study development (e.g., qualities of team members, budget for purchasing items) that are often not intentionally considered during the research process. Suggestions include adding a group component to the intervention to increase social interactions.

